# Supplementary material for: Pyruvate Dehydrogenase Kinase 1 inhibition mediated oxidative phosphorylation enhancement in cartilage promotes osteoarthritis progression
Source: BMC Musculoskelet Disord. 2023 Jul 20;24:597. doi: 10.1186/s12891-023-06585-6 (PMC10357736; doi:10.1186/s12891-023-06585-6)
Supplement: Supplementary file 1 — Additional file 1. [file 12891_2023_6585_MOESM1_ESM.zip › MF.Gsea.1653627316242/GOMF_PEPTIDE_HORMONE_BINDING.html]

Details for gene set GOMF\_PEPTIDE\_HORMONE\_BINDING[GSEA]

|  || Dataset | 55457\_collapsed\_to\_symbols.cls.cls #OA\_versus\_normal.cls.cls #OA\_versus\_normal\_repos |
| Phenotype | cls.cls#OA\_versus\_normal\_repos |
| Upregulated in class | normal |
| GeneSet | GOMF\_PEPTIDE\_HORMONE\_BINDING |
| Enrichment Score (ES) | -0.39710593 |
| Normalized Enrichment Score (NES) | -1.5115812 |
| Nominal p-value | 0.020477816 |
| FDR q-value | 0.17136654 |
| FWER p-Value | 1.0 |
Table: GSEA Results Summary

  

Fig 1: Enrichment plot: GOMF\_PEPTIDE\_HORMONE\_BINDING      
 Profile of the Running ES Score & Positions of GeneSet Members on the Rank Ordered List

  

| SYMBOL | TITLE | RANK IN GENE LIST | RANK METRIC SCORE | RUNNING ES | CORE ENRICHMENT || 1 | CRHBP | corticotropin releasing hormone binding protein [Source:HGNC Symbol;Acc:HGNC:2356] | 535 | 0.645 | -0.0022 | No |
| 2 | AVPR1B | arginine vasopressin receptor 1B [Source:HGNC Symbol;Acc:HGNC:896] | 865 | 0.552 | 0.0059 | No |
| 3 | CMKLR2 | chemerin chemokine-like receptor 2 [Source:HGNC Symbol;Acc:HGNC:4463] | 1439 | 0.437 | -0.0120 | No |
| 4 | CCKAR | cholecystokinin A receptor [Source:HGNC Symbol;Acc:HGNC:1570] | 1849 | 0.379 | -0.0207 | No |
| 5 | NPR3 | natriuretic peptide receptor 3 [Source:HGNC Symbol;Acc:HGNC:7945] | 1869 | 0.377 | 0.0009 | No |
| 6 | HCRTR1 | hypocretin receptor 1 [Source:HGNC Symbol;Acc:HGNC:4848] | 2644 | 0.286 | -0.0419 | No |
| 7 | GHRHR | growth hormone releasing hormone receptor [Source:HGNC Symbol;Acc:HGNC:4266] | 2721 | 0.278 | -0.0308 | No |
| 8 | GUCY2C | guanylate cyclase 2C [Source:HGNC Symbol;Acc:HGNC:4688] | 2982 | 0.252 | -0.0357 | No |
| 9 | VIPR1 | vasoactive intestinal peptide receptor 1 [Source:HGNC Symbol;Acc:HGNC:12694] | 3394 | 0.212 | -0.0547 | No |
| 10 | ACVR1 | activin A receptor type 1 [Source:HGNC Symbol;Acc:HGNC:171] | 3898 | 0.165 | -0.0838 | No |
| 11 | RAMP1 | receptor activity modifying protein 1 [Source:HGNC Symbol;Acc:HGNC:9843] | 4201 | 0.138 | -0.0989 | No |
| 12 | PRLR | prolactin receptor [Source:HGNC Symbol;Acc:HGNC:9446] | 4323 | 0.126 | -0.1005 | No |
| 13 | IDE | insulin degrading enzyme [Source:HGNC Symbol;Acc:HGNC:5381] | 5491 | 0.033 | -0.1893 | No |
| 14 | EBI3 | Epstein-Barr virus induced 3 [Source:HGNC Symbol;Acc:HGNC:3129] | 5532 | 0.030 | -0.1906 | No |
| 15 | MC3R | melanocortin 3 receptor [Source:HGNC Symbol;Acc:HGNC:6931] | 5910 | 0.002 | -0.2199 | No |
| 16 | MC4R | melanocortin 4 receptor [Source:HGNC Symbol;Acc:HGNC:6932] | 5918 | 0.001 | -0.2203 | No |
| 17 | CMKLR1 | chemerin chemokine-like receptor 1 [Source:HGNC Symbol;Acc:HGNC:2121] | 5980 | -0.003 | -0.2249 | No |
| 18 | CRHR1 | corticotropin releasing hormone receptor 1 [Source:HGNC Symbol;Acc:HGNC:2357] | 6390 | -0.034 | -0.2546 | No |
| 19 | CRHR2 | corticotropin releasing hormone receptor 2 [Source:HGNC Symbol;Acc:HGNC:2358] | 6672 | -0.054 | -0.2732 | No |
| 20 | GHR | growth hormone receptor [Source:HGNC Symbol;Acc:HGNC:4263] | 6740 | -0.058 | -0.2749 | No |
| 21 | NPR2 | natriuretic peptide receptor 2 [Source:HGNC Symbol;Acc:HGNC:7944] | 6771 | -0.061 | -0.2735 | No |
| 22 | GIPR | gastric inhibitory polypeptide receptor [Source:HGNC Symbol;Acc:HGNC:4271] | 6979 | -0.078 | -0.2848 | No |
| 23 | PTH1R | parathyroid hormone 1 receptor [Source:HGNC Symbol;Acc:HGNC:9608] | 7095 | -0.087 | -0.2884 | No |
| 24 | RAMP2 | receptor activity modifying protein 2 [Source:HGNC Symbol;Acc:HGNC:9844] | 7247 | -0.099 | -0.2941 | No |
| 25 | OXTR | oxytocin receptor [Source:HGNC Symbol;Acc:HGNC:8529] | 7534 | -0.121 | -0.3090 | No |
| 26 | GCGR | glucagon receptor [Source:HGNC Symbol;Acc:HGNC:4192] | 7697 | -0.134 | -0.3134 | No |
| 27 | GHSR | growth hormone secretagogue receptor [Source:HGNC Symbol;Acc:HGNC:4267] | 8472 | -0.191 | -0.3619 | No |
| 28 | LEPR | leptin receptor [Source:HGNC Symbol;Acc:HGNC:6554] | 8479 | -0.192 | -0.3507 | No |
| 29 | HCRTR2 | hypocretin receptor 2 [Source:HGNC Symbol;Acc:HGNC:4849] | 8495 | -0.193 | -0.3400 | No |
| 30 | GLP2R | glucagon like peptide 2 receptor [Source:HGNC Symbol;Acc:HGNC:4325] | 8606 | -0.202 | -0.3363 | No |
| 31 | PTH2R | parathyroid hormone 2 receptor [Source:HGNC Symbol;Acc:HGNC:9609] | 9197 | -0.247 | -0.3671 | No |
| 32 | CALCRL | calcitonin receptor like receptor [Source:HGNC Symbol;Acc:HGNC:16709] | 9584 | -0.282 | -0.3799 | Yes |
| 33 | NPR1 | natriuretic peptide receptor 1 [Source:HGNC Symbol;Acc:HGNC:7943] | 9634 | -0.286 | -0.3662 | Yes |
| 34 | GALR3 | galanin receptor 3 [Source:HGNC Symbol;Acc:HGNC:4134] | 9722 | -0.294 | -0.3551 | Yes |
| 35 | CALCR | calcitonin receptor [Source:HGNC Symbol;Acc:HGNC:1440] | 9937 | -0.315 | -0.3524 | Yes |
| 36 | VIPR2 | vasoactive intestinal peptide receptor 2 [Source:HGNC Symbol;Acc:HGNC:12695] | 10358 | -0.357 | -0.3633 | Yes |
| 37 | C2CD2L | C2CD2 like [Source:HGNC Symbol;Acc:HGNC:29000] | 10495 | -0.372 | -0.3512 | Yes |
| 38 | SCTR | secretin receptor [Source:HGNC Symbol;Acc:HGNC:10608] | 10726 | -0.396 | -0.3449 | Yes |
| 39 | CCKBR | cholecystokinin B receptor [Source:HGNC Symbol;Acc:HGNC:1571] | 10842 | -0.410 | -0.3288 | Yes |
| 40 | PIK3R1 | phosphoinositide-3-kinase regulatory subunit 1 [Source:HGNC Symbol;Acc:HGNC:8979] | 11099 | -0.443 | -0.3216 | Yes |
| 41 | INHBA | inhibin subunit beta A [Source:HGNC Symbol;Acc:HGNC:6066] | 11237 | -0.462 | -0.3041 | Yes |
| 42 | GALR1 | galanin receptor 1 [Source:HGNC Symbol;Acc:HGNC:4132] | 11851 | -0.560 | -0.3176 | Yes |
| 43 | GALR2 | galanin receptor 2 [Source:HGNC Symbol;Acc:HGNC:4133] | 12072 | -0.612 | -0.2973 | Yes |
| 44 | EDNRB | endothelin receptor type B [Source:HGNC Symbol;Acc:HGNC:3180] | 12075 | -0.613 | -0.2600 | Yes |
| 45 | ECE1 | endothelin converting enzyme 1 [Source:HGNC Symbol;Acc:HGNC:3146] | 12417 | -0.712 | -0.2430 | Yes |
| 46 | GLP1R | glucagon like peptide 1 receptor [Source:HGNC Symbol;Acc:HGNC:4324] | 12596 | -0.795 | -0.2082 | Yes |
| 47 | IGF1R | insulin like growth factor 1 receptor [Source:HGNC Symbol;Acc:HGNC:5465] | 12689 | -0.855 | -0.1632 | Yes |
| 48 | AVPR1A | arginine vasopressin receptor 1A [Source:HGNC Symbol;Acc:HGNC:895] | 12751 | -0.913 | -0.1121 | Yes |
| 49 | ADCYAP1R1 | ADCYAP receptor type I [Source:HGNC Symbol;Acc:HGNC:242] | 12790 | -0.952 | -0.0570 | Yes |
| 50 | INSR | insulin receptor [Source:HGNC Symbol;Acc:HGNC:6091] | 12856 | -1.071 | 0.0034 | Yes |
Table: GSEA details [plain text format]

  

Fig 2: GOMF\_PEPTIDE\_HORMONE\_BINDING      
 Blue-Pink O' Gram in the Space of the Analyzed GeneSet

  

Fig 3: GOMF\_PEPTIDE\_HORMONE\_BINDING: Random ES distribution      
 Gene set null distribution of ES for **GOMF\_PEPTIDE\_HORMONE\_BINDING**
